# Crystal structure and photoluminescence properties of *catena*-poly[[bis­(1-benzyl-1*H*-imidazole-κ*N* ^3^)cadmium(II)]-di-μ-azido-κ^4^ *N* ^1^:*N* ^3^]

**DOI:** 10.1107/S205698901901421X

**Published:** 2019-10-29

**Authors:** Ploy Assavajamroon, Filip Kielar, Kittipong Chainok, Nanthawat Wannarit

**Affiliations:** a Department of Chemistry, Faculty of Science and Technology, Thammasat University, Klong Luang, Pathum Thani 12121, Thailand; b Department of Chemistry, Faculty of Science, Naresuan University, Phitsanulok 65000, Thailand; c Materials and Textile Technology, Faculty of Science and Technology, Thammasat University, Klong Luang, Pathum Thani 12121, Thailand

**Keywords:** crystal structure, one-dimensional coordination polymer, cadmium(II), doubly-bridging azide ligand

## Abstract

The new title one-dimensional Cd^II^ coordination polymer, [Cd(C_10_H_10_N_2_)_2_(μ_1,3_-N_3_)_2_]_*n*_, has been synthesized and structurally characterized by single-crystal X-ray diffraction. The asymmetric unit consists of a Cd^II^ ion, one azide and one 1-benzyl­imidazole (bzi) ligand. The Cd^II^ ion is located on an inversion centre and is surrounded in a distorted octa­hedral coordination sphere by six N atoms from four symmetry-related azide ligands and two symmetry-related bzi ligands. The Cd^II^ ions are linked by double azide bridging ligands within a μ_1,3_-N_3_ end-to-end (EE) coordination mode, leading to a one-dimensional linear structure extending parallel to [100]. The supra­molecular framework is stabilized by the presence of weak C—H⋯N inter­actions, π–π stacking [centroid-to-centroid distance of 3.832 (2) Å] and C—H⋯π inter­actions between neighbouring chains.

## Abstract 2

The crystal structure and photoluminescence properties of a new one-dimensional Cd^II^ coordination polymer constructued by azide and 1-benzyl­imidazole (bzi) are reported.

## Chemical context   

Coordination polymers (CPs) have been receiving significant attention because of their inter­esting topologies (Zhang *et al.*, 2013 ▸), properties (Kitagawa *et al.*, 2004 ▸) and potential applications (He *et al.*, 2018 ▸; Gao *et al.*, 2019 ▸). Among various transition metal CPs, cadmium(II) coordination polymers containing nitro­gen-donor ligands have been widely investigated because of their potential applications in photoluminescence (PL) (Wang *et al.*, 2012 ▸) or photocatalysis (Wu *et al.*, 2017 ▸). Generally, the Cd^II^ ion adopts the stable [Kr]4*d* ^10^ electron configuration and its crystal chemistry is dominated by coordination numbers of four to six (Liu *et al.*, 2016 ▸). As for the choice of nitro­gen-donor ligands, pseudohalides in the form of azide (N_3_^−^), thio­cyanate (NCS^−^) or dicyanamide (N(CN)_2_^−^) are good candidates as anionic linkers (Mautner *et al.*, 2019 ▸). In particular, the azide ligand is an attractive bridging ligand due to the variability of its coordination modes, such as the common μ_1,1_ (end-on, EO) and μ_1,3_ (end-to-end, EE) mode with single or double azide bridges (Ribas *et al.*, 1999 ▸). Therefore, such ligands are used for studying magnetochemistry and for the construction of coordination frameworks. Imidazole-based derivatives with aromatic substituents, for example, 1-benzyl­imidazole (bzi) (Krinchampa *et al.*, 2016 ▸) or 1,4-bis­(imidazol-1-ylmeth­yl)benzene (bix) (Adarsh *et al.*, 2016 ▸), are usually selected for extending the structural dimensions and increasing the photoluminescence properties of their CPs due to the existence of supra­molecular inter­actions in terms of hydrogen bonds, π–π stacking and/or C—H⋯π to increase the rigidity and framework stabilities. To the best of our knowledge, the number of Cd^II^ coordination polymers with mixed nitro­gen-donor ligands, *e.g.* azide and bzi ligands, is still limited. As part of our ongoing exploration of new members of *d* ^10^ CPs and investigation of their properties (Krinchampa *et al.*, 2016 ▸; Sangsawang *et al.*, 2017 ▸), a family of Cd^II^ coordination polymers containing mixed nitro­gen-donor ligands, *i.e.* bzi and pseudohalide ligands, such as azide (N_3_^−^), thio­cyanate (NCS^−^) and dicyanamide (N(CN)_2_^−^), have been designed and prepared. In this work, a new one-dimensional Cd^II^ coordination polymer, [Cd(bzi)_2_(μ_1,3_-N_3_)_2_]_*n*_, was synthesized and characterized. Details of the synthesis, crystal structure determination and photoluminescence properties of this com­pound are reported herein.

## Structural commentary   

**Figure 1:**
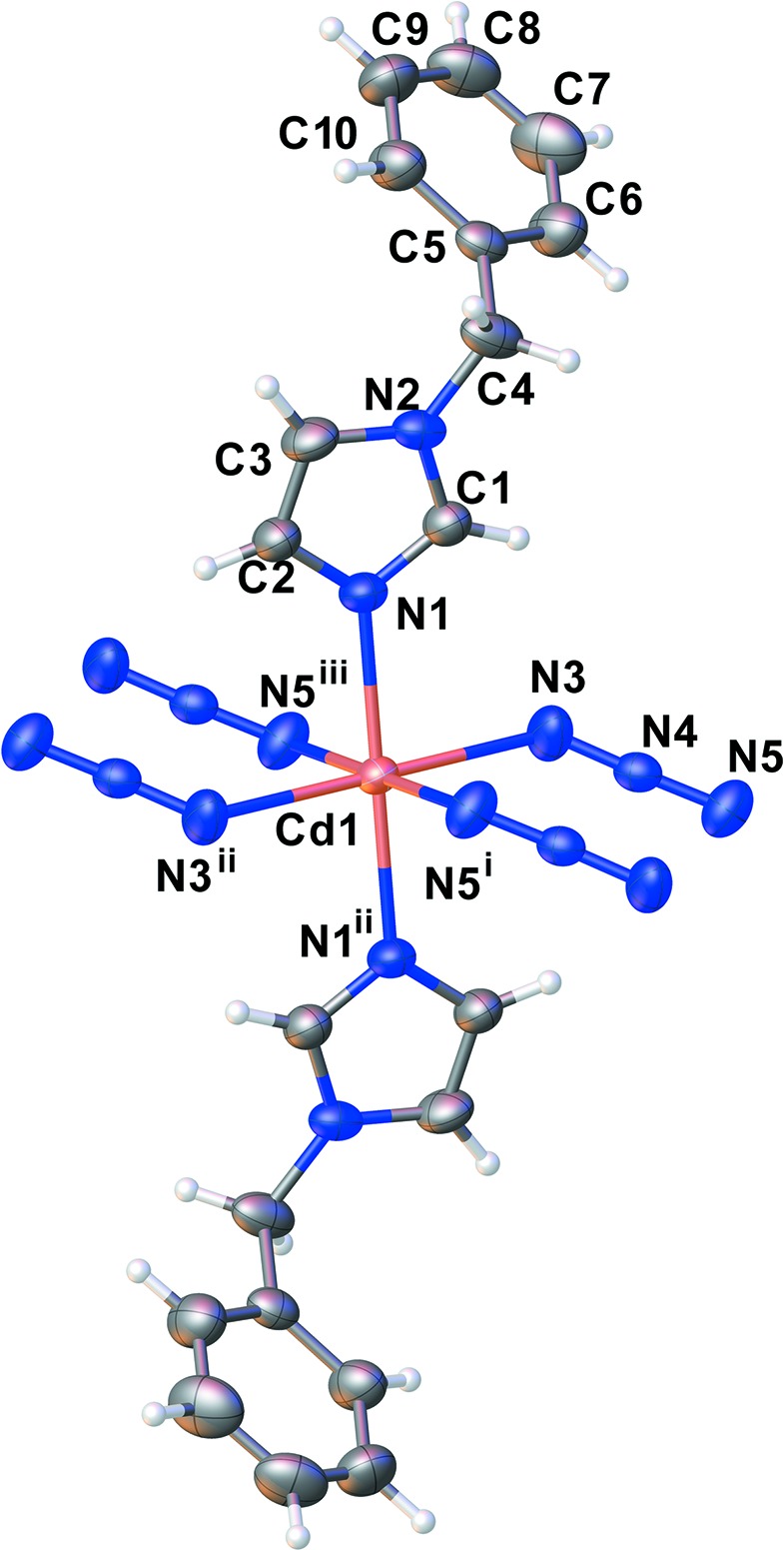
The coordination environment of Cd^II^, with displacement ellipsoids drawn at the 50% probability level. [Symmetry codes: (i) −*x*, −*y* + 2, −*z* + 1; (ii) −*x* + 1, −*y* + 2, −*z* + 1; (iii) *x* + 1, +*y*, +*z*.]

**Figure 2:**
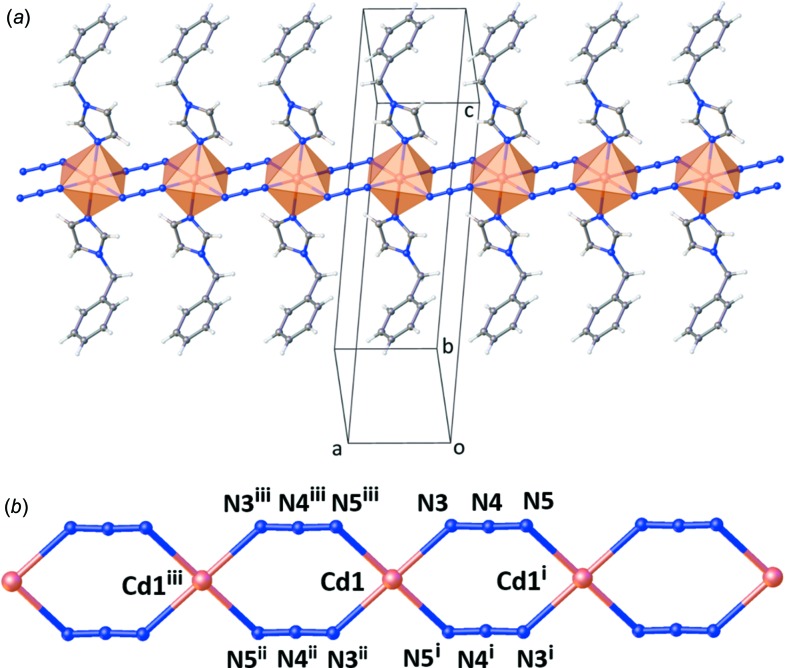
Views of the crystal structure of the title com­pound, emphasizing (*a*) the one-dimensional linear doubly-bridged chain-like structure and (*b*) the doubly-bridged (EE) azide coordination mode (bzi ligands have been omitted for clarity). [Symmetry codes: (i) −*x*, −*y* + 2, −*z* + 1; (ii) −*x* + 1, −*y* + 2, −*z* + 1; (iii) *x* + 1, +*y*, +*z*.]

The asymmetric unit of the title com­pound consists of a Cd^II^ ion (site symmetry 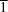), one azide ligand and one bzi ligand (Fig. 1 ▸). The distorted octa­hedral coordination environment of the Cd^II^ ion is defined by six N atoms. Two are from two symmetry-related bzi ligands in the axial positions with the shortest Cd—N distance, and four are from four symmetry-related azide ligands in equatorial positions with slightly larger distances; angular distortions are small (Table 1 ▸). Neighbouring Cd^II^ ions are linked by doubly end-to-end (EE) binding azide bridges, resulting in a one-dimensional linear chain-like structure extending along [100] (Fig. 2 ▸). The Cd⋯Cd distance in the chain is 5.5447 (3) Å, which is longer than in a previously reported one-dimensional zigzag chain-like structure of a Cd^II^ coordination polymer, [Cd(N_3_)_2_(3,5-DMP)_2_] (Goher *et al.*, 2003 ▸).

## Supra­molecular features   

**Figure 3:**
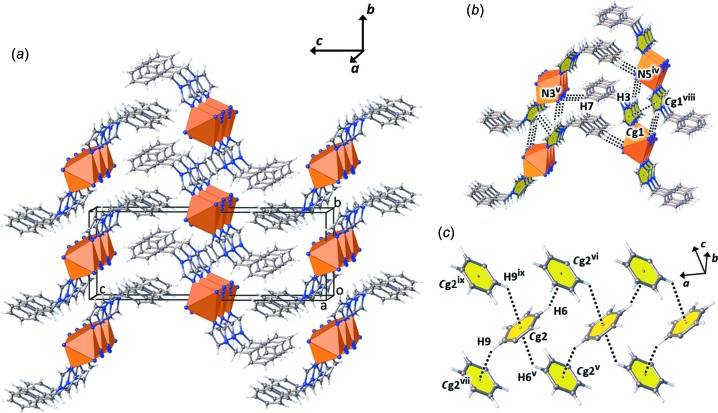
Views of (*a*) the crystal packing, (*b*) the C—H⋯N hydrogen bonding and π–π inter­actions, and (*c*) the weak inter­molecular C—H⋯π inter­actions between adjacent chains of the title com­pound. [Symmetry codes (iv) *x* + 1, *y* − 1, +*z*; (v) −*x* + 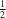, *y* − 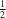, −*z* + 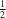; (vi) −*x* + 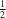, *y* + 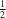, −*z* + 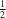; (vii) −*x* + 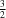, *y* − 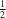, −*z* + 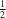; (viii) −*x* + 1, −*y* + 1, −*z* + 1; (ix) −*x* + 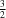, *y* + 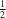, −*z* + 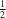.]

The crystal structure of the title com­pound is stabilized by various weak inter­actions, including C—H⋯N hydrogen bonding, π–π stacking and inter­molecular C—H⋯π inter­actions between adjacent chains (Fig. 3 ▸ *a*). Hydrogen-bonding inter­actions are found between the C—H groups of the phenyl rings and the N atoms of the azide bridging ligands (Table 2 ▸ and Fig. 3 ▸ *b*); π–π stacking between adjacent chains is associated with the symmetry-related imidazole rings of the bzi ligands [*Cg*1⋯*Cg*1(−*x* + 1, −*y* + 1, −*z* + 1) = 3.832 (2) Å; slippage = 1.477 Å; inter­planar distance = 3.536 (3) Å; *Cg*1 is the centroid of the imidazole N1/C1/N2/C2/C3 ring], as shown in Fig. 3 ▸(*b*). Moreover, C—H⋯π inter­actions between the phenyl rings of the bzi ligands of different chains are observed (Fig. 3 ▸ *c* and Table 2 ▸).
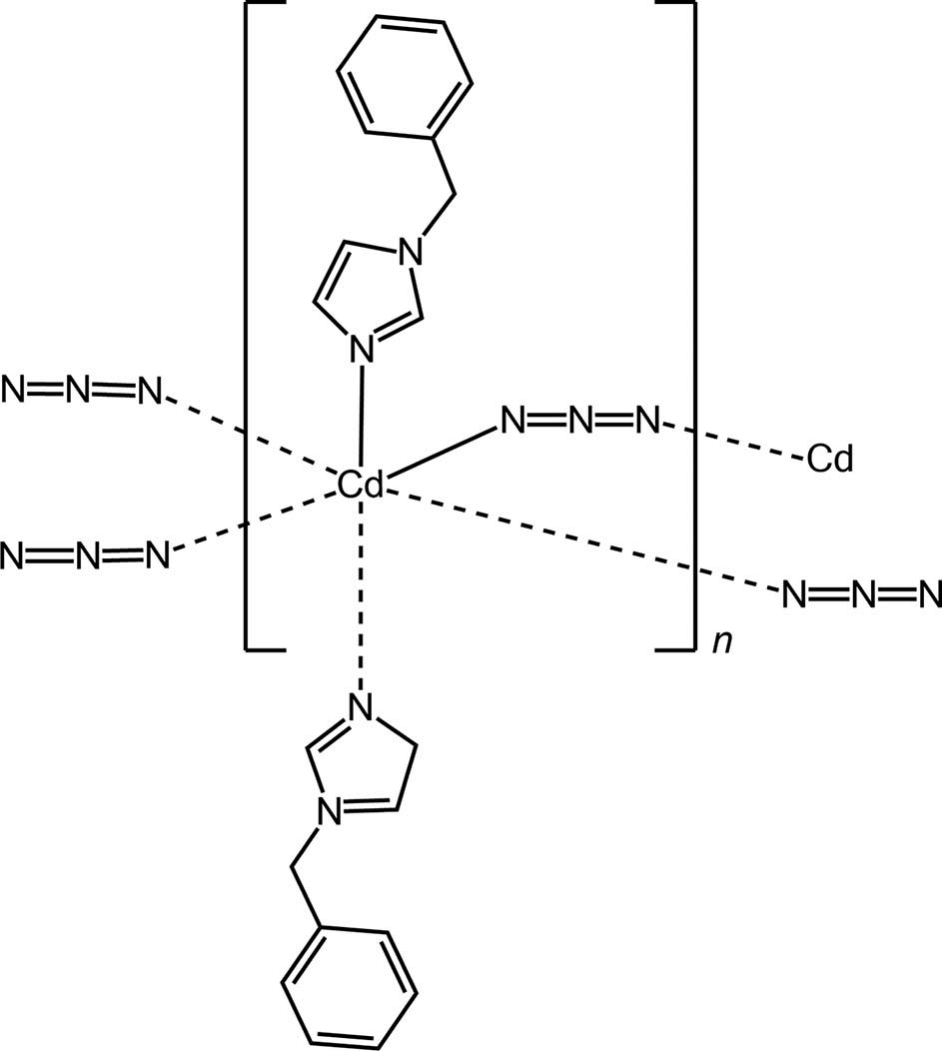


## Characterization   

The FT–IR spectrum (Fig. S1 in the supporting information) of the title com­pound presents the characteristic bands of the N_3_^−^ ligand at 2058 cm^−1^ and the characteristic bands of the bzi ligand including C—H aromatic stretching at 3113–3028 cm^−1^, C=N and C=C stretching at 1602–1506 cm^−1^, C—C stretching at 1396 cm^−1^ and C—N stretching at 1233 cm^−1^. The IR spectrum also reveals a band at 3365 cm^−1^, indicating the N⋯H hydrogen-bonding inter­action in this com­pound.

Plots of the experimental and simulated powder X-ray diffraction (PXRD) patterns of the title com­pound are shown in Fig. S2 of the supporting information, revealing a good match and thus phase purity and repeatable synthesis.

The thermal stability of the title com­pound has been investigated by means of thermogravimetric analysis from room temperature to 1073 K under a nitro­gen atmosphere. Based on the results (Fig. S3 in the supporting information), the structure of the title com­pound is stable up to around 470 K. Above this temperature, the structure starts to collapse by losing a mass percentage of 57.7%, which corresponds to the loss of two bzi ligands. The second step of mass loss by about 30.6% corresponds to the loss of the remaining azide ligands. Further increasing the temperature leads only to a slight increase of the mass loss until CdO was formed as the final product.

## Photoluminescence (PL) properties   

**Figure 4:**
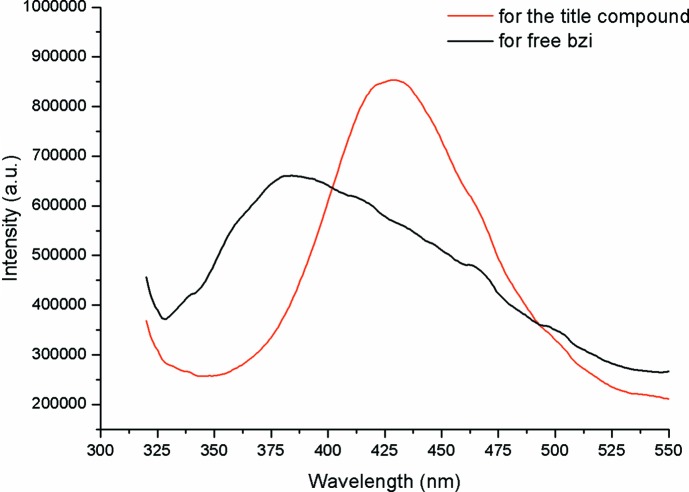
The solid-state PL spectra of the title com­pound (red line) and free bzi (black line).

Fig. 4 ▸ presents the solid-state PL emission spectra of the free bzi ligand and the title com­pound. It should be noted that the signal in the emission spectra below 330 nm belongs to the tail of the scattered excitation light. The PL spectrum of the free bzi ligand reveals a broad band with the centre at 384 nm (λ_ex_ = 305 nm), which is assigned to the π→π* and *n*→π* transitions of the delocalized electrons within the aromatic phenyl and imidazole rings. Inter­estingly, the emission spectrum of the title com­pound exhibits a red shift with a λ_max_ of 429 nm (λ_ex_ = 305 nm) and a higher emission intensity in com­parison with that of free bzi. Furthermore, the emission peak of the title com­pound is less broad than that of the bzi ligand. The PL features of the title com­pound can be attributed to ligand-to-metal charge transfer (LMCT). The increased intensity is presumably caused by the increased rigidity for the bzi ligands due to the presence of numerous weak supra­molecular inter­actions between the chains in the crystal structure. This increased rigidity likely enhances the emission properties of the title com­pound due to limiting the probability of non­radiative decay of the excited state.

## Database survey   

One-dimensional linear chain-like Cd^II^ coordination polymers constructed by one type of doubly end-to-end (EE) bound azide bridges and ligands based on imidazole derivatives are rare in the literature. To the best of our knowledge, there are only a few first-row transition metal coordination polymers constructed by μ_1,3_-N_3_^−^ and differently substituted pyridine derivatives: [Cu(N_3_)_2_(*L*1)_2_]_*n*_ [Cambridge Structural Database (CSD; Groom *et al.*, 2016 ▸) refcode LOYROG; Dalai *et al.*, 2002 ▸], [Co(N_3_)_2_(bepy)_2_]_*n*_ (TUJCEI; Zhao *et al.*, 2015 ▸) and [Mn(N_3_)_2_(*L*2)_2_]_*n*_ (CEMTOG; Khani *et al.*, 2018 ▸) {where *L*1 = 4-(di­methyl­amino)­pyridine, bepy = 4-benzyl­pyridine and *L*2 = *N*′-[4-(di­methyl­amino)­benzyl­idene]isonicotinohydrazide}. On the other hand, previously reported CPs containing μ_1,3_-N_3_^−^ and 3,5-di­methyl­pyridine (3,5-DMP) ligands in [*M*(N_3_)_2_(3,5-DMP)_2_] [*M* = Cd (EHEYIZ; Goher *et al.*, 2003 ▸) and Ni (LEWMAD; Lu *et al.*, 2012 ▸)] exhibit one-dimensional structures with zigzag chains.

## Synthesis and crystallization   

A methano­lic solution (5 ml) of bzi (1.0 mmol) was introduced slowly to a methano­lic solution (5 ml) of Cd(NO_3_)_2_·4H_2_O (1.0 mmol). A DMSO solution (5 ml) of NaN_3_ (2.0 mmol) was then added slowly to the mixed solution, resulting in the immediate formation of a white precipitate. The precipitate was dropped slowly into a DMSO–DMF (1:2 *v*/*v*) mixture (9 ml) under continuous stirring at 333 K over a period of 30 min, and was kept stirring until the solution became clear. Finally, the solution was filtered and allowed to slowly evaporate in air at room temperature. Colourless crystals of the title com­pound were obtained within 3 d (yield 23.36%, 119.80 mg, based on the Cd^II^ salt). Elemental analysis calculated (found) (%) for C_20_H_20_CdN_10_: C 46.84 (46.83), H 3.9 3(3.62), N 27.31 (27.05). IR (KBr, cm^−1^): 3370 (*m*), 3109 (*s*), 2062 (*s*, broad), 1612 (*w*), 1510 (*m*), 1440 (*m*), 1395 (*w*), 1355 (*m*), 1280 (*m*), 1233 (*m*), 1098 (*s*), 1030 (*m*), 942 (*m*), 822 (*m*), 767 (*m*), 712 (*s*), 652 (*m*, 625 (*m*), 462 (*w*).

## Refinement   

The crystal data, data collection and structure refinement details are summarized in Table 3 ▸. All H atoms were generated geometrically and refined using a riding model, with C—H = 0.93 Å and *U*_iso_(H) = 1.2*U*_eq_(C).

## Supplementary Material

Crystal structure: contains datablock(s) I, global. DOI: 10.1107/S205698901901421X/wm5527sup1.cif

Structure factors: contains datablock(s) I. DOI: 10.1107/S205698901901421X/wm5527Isup2.hkl

Supporting information file. DOI: 10.1107/S205698901901421X/wm5527sup3.pdf

CCDC references: 1959895, 1959895

Additional supporting information:  crystallographic information; 3D view; checkCIF report

## Figures and Tables

**Table 1:** Selected geometric parameters (Å, °)

Cd1—N1	2.2834 (19)	Cd1—N5^i^	2.400 (2)
Cd1—N3	2.346 (2)		
			
N1^ii^—Cd1—N3	92.30 (8)	N3—Cd1—N5^iii^	91.87 (9)
N1—Cd1—N3	87.70 (8)	N3—Cd1—N5^i^	88.13 (9)
N1—Cd1—N5^iii^	90.74 (8)	N1—Cd1—N5^i^	89.26 (8)

Symmetry codes: (i) 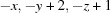; (ii) 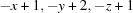; (iii) 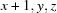.

**Table 2:** Hydrogen-bond geometry (Å, °) *Cg*2 is the centroid of the C5–C10 ring.

*D*—H⋯*A*	*D*—H	H⋯*A*	*D*⋯*A*	*D*—H⋯*A*
C3—H3⋯N5^iv^	0.93	2.49 (1)	3.313 (4)	148 (1)
C7—H7⋯N3^v^	0.93	2.62 (1)	3.368 (4)	138 (1)
C6—H6⋯*Cg*2^vi^	0.93	3.17 (1)	3.890 (3)	135 (1)
C9—H9⋯*Cg*2^vii^	0.93	3.10 (1)	3.833 (3)	138 (1)

Symmetry codes: (iv) 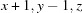; (v) 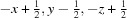; (vi) 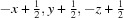; (vii) 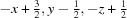.

**Table 3:** Experimental details

Crystal data	
Chemical formula	[Cd(C_10_H_10_N_2_)_2_(N_3_)_2_]
*M*_r_	512.86
Crystal system, space group	Monoclinic, *P*2_1_/*n*
Temperature (K)	296
*a*, *b*, *c* (Å)	5.5447 (3), 8.4301 (4), 22.9517 (11)
β (°)	90.351 (2)
*V* (Å^3^)	1072.80 (9)
*Z*	2
Radiation type	Mo *K*α
μ (mm^−1^)	1.05
Crystal size (mm)	0.32 × 0.3 × 0.22

Data collection	
Diffractometer	Bruker D8 QUEST CMOS PHOTON II
Absorption correction	Multi-scan (*SADABS*; Krause *et al.*, 2015 ▸)
*T*_min_, *T*_max_	0.712, 0.746
No. of measured, independent and observed [*I* > 2σ(*I*)] reflections	42354, 3817, 2858
*R*_int_	0.072
(sin θ/λ)_max_ (Å^−1^)	0.751

Refinement	
*R*[*F* ^2^ > 2σ(*F* ^2^)], *wR*(*F* ^2^), *S*	0.043, 0.080, 1.12
No. of reflections	3817
No. of parameters	143
H-atom treatment	H-atom parameters constrained
Δρ_max_, Δρ_min_ (e Å^−3^)	0.49, −0.43

Computer programs: *APEX3* (Bruker, 2016 ▸), *SAINT* (Bruker, 2016 ▸), *SHELXT* (Sheldrick, 2015*a* ▸), *SHELXL2017* (Sheldrick, 2015*b* ▸) and *OLEX2* (Dolomanov *et al.*, 2009 ▸).

## supplementary crystallographic information

### Crystal data

**Table:**

[Cd(C_10_H_10_N_2_)_2_(N_3_)_2_]	*F*(000) = 516
*M**_r_* = 512.86	*D*_x_ = 1.588 Mg m^−^^3^
Monoclinic, *P*2_1_/*n*	Mo *K*α radiation, λ = 0.71073 Å
*a* = 5.5447 (3) Å	Cell parameters from 9996 reflections
*b* = 8.4301 (4) Å	θ = 3.0–32.3°
*c* = 22.9517 (11) Å	µ = 1.05 mm^−^^1^
β = 90.351 (2)°	*T* = 296 K
*V* = 1072.80 (9) Å^3^	Block, colourless
*Z* = 2	0.32 × 0.3 × 0.22 mm

### Data collection

**Table:**

Bruker D8 QUEST CMOS PHOTON II diffractometer	3817 independent reflections
Radiation source: sealed x-ray tube, Mo	2858 reflections with *I* > 2σ(*I*)
Graphite monochromator	*R*_int_ = 0.072
Detector resolution: 7.39 pixels mm^-1^	θ_max_ = 32.3°, θ_min_ = 3.0°
ω and φ scans	*h* = −8→8
Absorption correction: multi-scan (SADABS; Krause *et al*., 2015)	*k* = −12→12
*T*_min_ = 0.712, *T*_max_ = 0.746	*l* = −34→34
42354 measured reflections	

### Refinement

**Table:**

Refinement on *F*^2^	Hydrogen site location: inferred from neighbouring sites
Least-squares matrix: full	H-atom parameters constrained
*R*[*F*^2^ > 2σ(*F*^2^)] = 0.043	*w* = 1/[σ^2^(*F*_o_^2^) + (0.0237*P*)^2^ + 0.7897*P*] where *P* = (*F*_o_^2^ + 2*F*_c_^2^)/3
*wR*(*F*^2^) = 0.080	(Δ/σ)_max_ < 0.001
*S* = 1.12	Δρ_max_ = 0.49 e Å^−^^3^
3817 reflections	Δρ_min_ = −0.43 e Å^−^^3^
143 parameters	Extinction correction: SHELXL2017 (Sheldrick, 2015b), Fc^*^=kFc[1+0.001xFc^2^λ^3^/sin(2θ)]^-1/4^
0 restraints	Extinction coefficient: 0.0043 (7)
Primary atom site location: dual	

### Special details

**Table:**

Geometry. All esds (except the esd in the dihedral angle between two l.s. planes) are estimated using the full covariance matrix. The cell esds are taken into account individually in the estimation of esds in distances, angles and torsion angles; correlations between esds in cell parameters are only used when they are defined by crystal symmetry. An approximate (isotropic) treatment of cell esds is used for estimating esds involving l.s. planes.

### Fractional atomic coordinates and isotropic or equivalent isotropic displacement parameters (Å^2^)

**Table:**

	*x*	*y*	*z*	*U*_iso_*/*U*_eq_	
Cd1	0.500000	1.000000	0.500000	0.03071 (9)	
N1	0.5662 (4)	0.7657 (2)	0.45279 (9)	0.0364 (4)	
N2	0.4732 (4)	0.5441 (2)	0.40781 (9)	0.0361 (5)	
N3	0.1837 (4)	1.0467 (3)	0.43386 (10)	0.0495 (6)	
N4	−0.0153 (4)	1.0885 (2)	0.43738 (8)	0.0293 (4)	
N5	−0.2152 (4)	1.1320 (3)	0.43834 (10)	0.0442 (5)	
C1	0.4075 (5)	0.6938 (3)	0.41923 (11)	0.0391 (5)	
H1	0.267175	0.740956	0.405159	0.047*	
C2	0.7433 (5)	0.6557 (3)	0.46316 (11)	0.0417 (6)	
H2	0.880614	0.672494	0.485806	0.050*	
C3	0.6881 (5)	0.5197 (3)	0.43559 (12)	0.0451 (6)	
H3	0.778880	0.426908	0.435479	0.054*	
C4	0.3379 (5)	0.4268 (4)	0.37310 (12)	0.0481 (7)	
H4A	0.168657	0.455917	0.372746	0.058*	
H4B	0.351963	0.323822	0.391634	0.058*	
C5	0.4247 (5)	0.4141 (3)	0.31116 (10)	0.0366 (5)	
C6	0.3045 (6)	0.4900 (4)	0.26668 (14)	0.0563 (7)	
H6	0.168444	0.550339	0.274880	0.068*	
C7	0.3837 (8)	0.4777 (4)	0.20985 (14)	0.0694 (10)	
H7	0.300687	0.529653	0.180124	0.083*	
C8	0.5824 (7)	0.3901 (4)	0.19727 (13)	0.0618 (9)	
H8	0.637397	0.383724	0.159140	0.074*	
C9	0.7007 (6)	0.3116 (4)	0.24067 (14)	0.0605 (8)	
H9	0.835053	0.250246	0.231895	0.073*	
C10	0.6230 (6)	0.3221 (4)	0.29772 (12)	0.0503 (7)	
H10	0.704249	0.267304	0.327012	0.060*	

### Atomic displacement parameters (Å^2^)

**Table:**

	*U*^11^	*U*^22^	*U*^33^	*U*^12^	*U*^13^	*U*^23^
Cd1	0.02939 (12)	0.03079 (13)	0.03194 (13)	0.00640 (11)	−0.00134 (8)	−0.00695 (11)
N1	0.0393 (11)	0.0338 (11)	0.0359 (10)	0.0070 (9)	−0.0021 (8)	−0.0085 (9)
N2	0.0453 (12)	0.0304 (10)	0.0328 (10)	−0.0014 (8)	0.0040 (9)	−0.0062 (8)
N3	0.0389 (12)	0.0709 (16)	0.0385 (11)	0.0148 (11)	−0.0086 (9)	−0.0046 (11)
N4	0.0399 (11)	0.0245 (9)	0.0236 (9)	0.0024 (8)	−0.0025 (8)	0.0007 (7)
N5	0.0389 (12)	0.0437 (13)	0.0501 (13)	0.0104 (10)	0.0068 (10)	0.0092 (10)
C1	0.0395 (13)	0.0402 (14)	0.0376 (13)	0.0078 (11)	−0.0021 (10)	−0.0057 (11)
C2	0.0405 (14)	0.0421 (14)	0.0425 (14)	0.0103 (11)	−0.0040 (11)	−0.0093 (11)
C3	0.0550 (15)	0.0334 (15)	0.0469 (14)	0.0140 (12)	−0.0008 (12)	−0.0056 (11)
C4	0.0564 (17)	0.0459 (16)	0.0420 (14)	−0.0187 (13)	0.0112 (13)	−0.0122 (12)
C5	0.0431 (13)	0.0329 (13)	0.0337 (12)	−0.0083 (10)	0.0004 (10)	−0.0065 (10)
C6	0.0616 (18)	0.0559 (18)	0.0513 (16)	0.0155 (16)	−0.0086 (13)	−0.0052 (15)
C7	0.101 (3)	0.064 (2)	0.0436 (16)	0.009 (2)	−0.0145 (17)	0.0064 (15)
C8	0.085 (2)	0.066 (2)	0.0339 (14)	−0.0078 (19)	0.0064 (15)	−0.0049 (14)
C9	0.0557 (19)	0.072 (2)	0.0537 (18)	0.0121 (16)	0.0060 (14)	−0.0170 (16)
C10	0.0584 (18)	0.0533 (17)	0.0393 (14)	0.0120 (14)	−0.0068 (12)	−0.0048 (13)

### Geometric parameters (Å, º)

**Table:**

Cd1—N1	2.2834 (19)	C3—H3	0.9300
Cd1—N1^i^	2.2834 (19)	C4—H4A	0.9700
Cd1—N3	2.346 (2)	C4—H4B	0.9700
Cd1—N3^i^	2.346 (2)	C4—C5	1.507 (3)
Cd1—N5^ii^	2.400 (2)	C5—C6	1.374 (4)
Cd1—N5^iii^	2.400 (2)	C5—C10	1.382 (4)
N1—C1	1.314 (3)	C6—H6	0.9300
N1—C2	1.371 (3)	C6—C7	1.383 (5)
N2—C1	1.340 (3)	C7—H7	0.9300
N2—C3	1.364 (4)	C7—C8	1.359 (5)
N2—C4	1.472 (3)	C8—H8	0.9300
N3—N4	1.161 (3)	C8—C9	1.361 (5)
N4—N5	1.168 (3)	C9—H9	0.9300
C1—H1	0.9300	C9—C10	1.384 (4)
C2—H2	0.9300	C10—H10	0.9300
C2—C3	1.344 (4)		
			
N1—Cd1—N1^i^	180.0	C3—C2—H2	125.2
N1^i^—Cd1—N3^i^	87.70 (8)	N2—C3—H3	126.7
N1^i^—Cd1—N3	92.30 (8)	C2—C3—N2	106.7 (2)
N1—Cd1—N3^i^	92.30 (8)	C2—C3—H3	126.7
N1—Cd1—N3	87.70 (8)	N2—C4—H4A	108.9
N1^i^—Cd1—N5^ii^	90.73 (8)	N2—C4—H4B	108.9
N1^i^—Cd1—N5^iii^	89.27 (8)	N2—C4—C5	113.2 (2)
N1—Cd1—N5^iii^	90.74 (8)	H4A—C4—H4B	107.8
N1—Cd1—N5^ii^	89.26 (8)	C5—C4—H4A	108.9
N3^i^—Cd1—N3	180.0	C5—C4—H4B	108.9
N3—Cd1—N5^iii^	91.87 (9)	C6—C5—C4	120.8 (3)
N3^i^—Cd1—N5^iii^	88.13 (9)	C6—C5—C10	118.6 (3)
N3—Cd1—N5^ii^	88.13 (9)	C10—C5—C4	120.6 (3)
N3^i^—Cd1—N5^ii^	91.87 (9)	C5—C6—H6	119.6
N5^ii^—Cd1—N5^iii^	180.0	C5—C6—C7	120.7 (3)
C1—N1—Cd1	124.62 (16)	C7—C6—H6	119.6
C1—N1—C2	105.4 (2)	C6—C7—H7	119.9
C2—N1—Cd1	128.34 (17)	C8—C7—C6	120.2 (3)
C1—N2—C3	106.8 (2)	C8—C7—H7	119.9
C1—N2—C4	126.9 (2)	C7—C8—H8	120.1
C3—N2—C4	126.3 (2)	C7—C8—C9	119.8 (3)
N4—N3—Cd1	135.45 (18)	C9—C8—H8	120.1
N3—N4—N5	177.0 (2)	C8—C9—H9	119.7
N4—N5—Cd1^iv^	119.56 (17)	C8—C9—C10	120.7 (3)
N1—C1—N2	111.6 (2)	C10—C9—H9	119.7
N1—C1—H1	124.2	C5—C10—C9	120.0 (3)
N2—C1—H1	124.2	C5—C10—H10	120.0
N1—C2—H2	125.2	C9—C10—H10	120.0
C3—C2—N1	109.6 (2)		
			
Cd1—N1—C1—N2	−166.25 (16)	C4—N2—C1—N1	178.4 (2)
Cd1—N1—C2—C3	165.85 (18)	C4—N2—C3—C2	−178.3 (2)
N1—C2—C3—N2	−0.3 (3)	C4—C5—C6—C7	−179.7 (3)
N2—C4—C5—C6	−99.6 (3)	C4—C5—C10—C9	−180.0 (3)
N2—C4—C5—C10	82.3 (3)	C5—C6—C7—C8	0.0 (5)
C1—N1—C2—C3	0.1 (3)	C6—C5—C10—C9	1.8 (4)
C1—N2—C3—C2	0.4 (3)	C6—C7—C8—C9	1.3 (6)
C1—N2—C4—C5	98.1 (3)	C7—C8—C9—C10	−1.0 (5)
C2—N1—C1—N2	0.2 (3)	C8—C9—C10—C5	−0.5 (5)
C3—N2—C1—N1	−0.4 (3)	C10—C5—C6—C7	−1.5 (5)
C3—N2—C4—C5	−83.3 (4)		

Symmetry codes: (i) −*x*+1, −*y*+2, −*z*+1; (ii) −*x*, −*y*+2, −*z*+1; (iii) *x*+1, *y*, *z*; (iv) *x*−1, *y*, *z*.

### Hydrogen-bond geometry (Å, º)

**Table:**

*D*—H···*A*	*D*—H	H···*A*	*D*···*A*	*D*—H···*A*
C3—H3···N5^v^	0.93	2.49 (1)	3.313 (4)	148 (1)
C7—H7···N3^vi^	0.93	2.62 (1)	3.368 (4)	138 (1)
C6—H6···*Cg*2^vii^	0.93	3.17 (1)	3.890 (3)	135 (1)
C9—H9···*Cg*2^viii^	0.93	3.10 (1)	3.833 (3)	138 (1)

Symmetry codes: (v) *x*+1, *y*−1, *z*; (vi) −*x*+1/2, *y*−1/2, −*z*+1/2; (vii) −*x*+1/2, *y*+1/2, −*z*+1/2; (viii) −*x*+3/2, *y*−1/2, −*z*+1/2.
