# Supplementary material for: Crystal structure and photoluminescence properties of catena-poly[[bis­(1-benzyl-1H-imidazole-κN 3)cadmium(II)]-di-μ-azido-κ4 N 1:N 3]
Source: Acta Crystallogr E Crystallogr Commun. 2019 Oct 29;75(Pt 11):1748–52. doi: 10.1107/S205698901901421X (PMC6829744; doi:10.1107/S205698901901421X)
Supplement: Supplementary file 3 [file e-75-01748-sup3.pdf]

Supporting Information

Crystal structure and photoluminescent properties of *catena*-poly[[bis(1-benzyl-1*H*-imidazole-κN^3)cadmium(II)]-di-μ-azido-$\kappa^2 N^1$:N^3]

Ploy Assavajamroon,[a] Filip Kielar,[b] Kittipong Chainok[c] and Nanthawat Wannarit[a]*

[a] Department of Chemistry, Faculty of Science and Technology, Thammasat University, Klong Luang, Pathum Thani 12121, Thailand

[b] Department of Chemistry, Faculty of Science, Naresuan University, Phitsanulok, 65000, Thailand

[c] Materials and Textile Technology, Faculty of Science and Technology, Thammasat University, Klong Luang, Pathum Thani 12121, Thailand

Correspondence email: nwan0110@tu.ac.th

Figure S1

FT-IR spectrum of the title compound

Figure S2

PXRD patterns of the title compound

Figure S3

TGA curve of the title compound

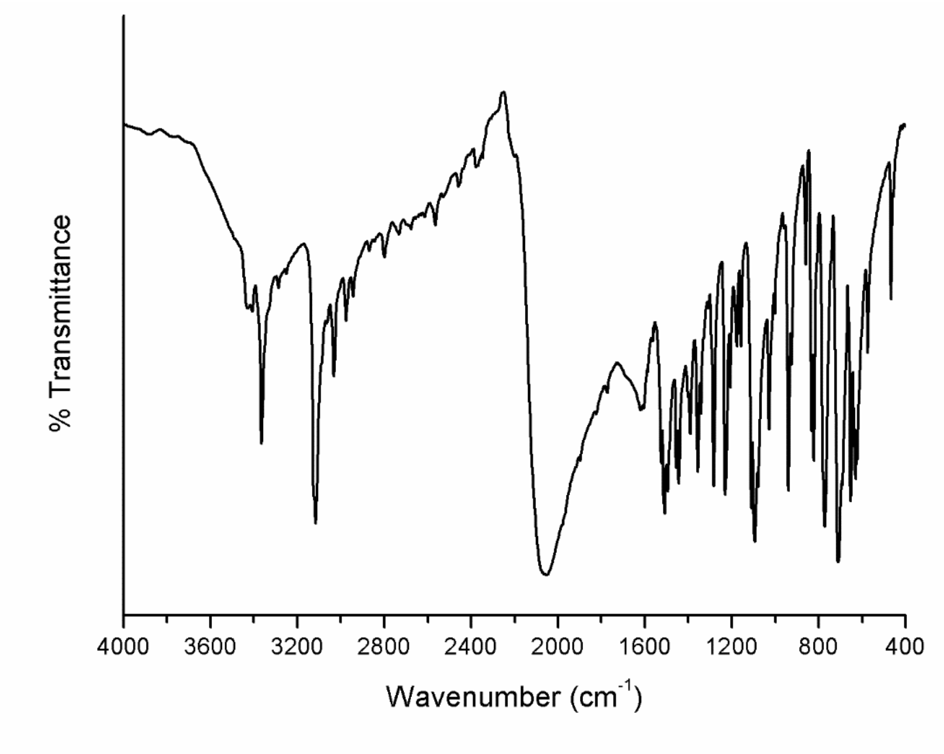

Figure S1

FT-IR spectrum of the title compound

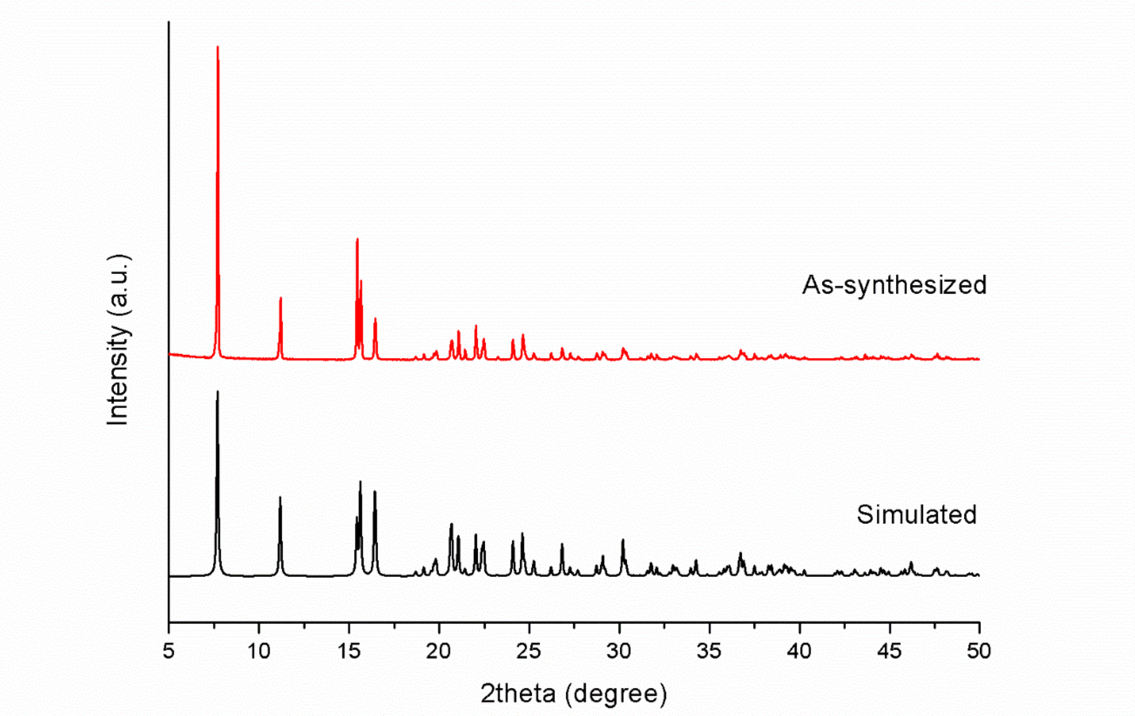

Figure S2

PXRD patterns of the title compound

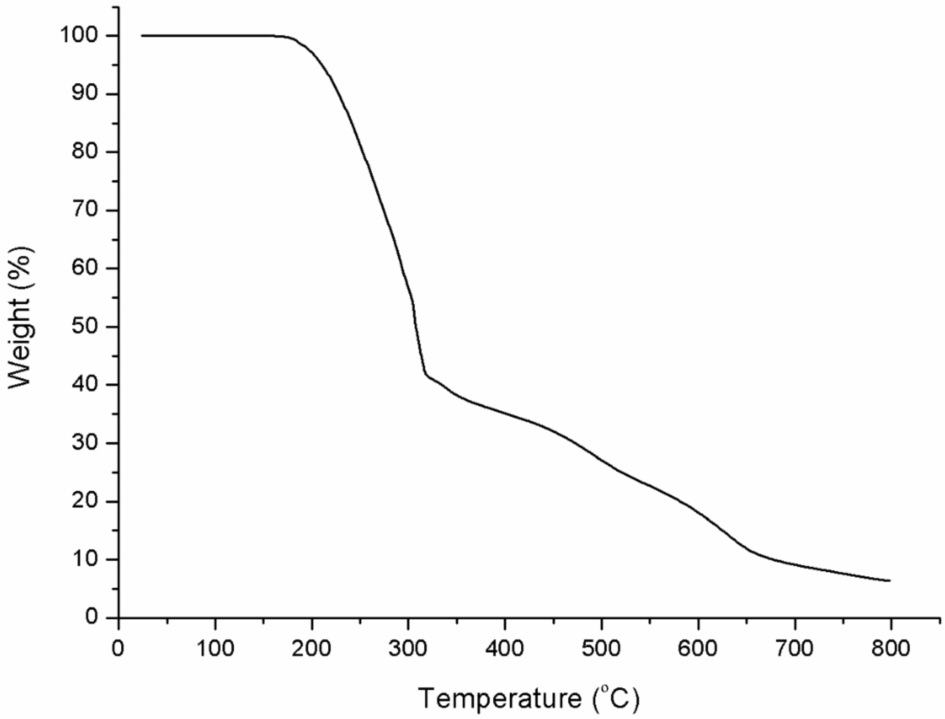

Figure S3

TGA curve of the title compound
